# Hospital Meetings, &c.

**Published:** 1899-03-25

**Authors:** 


### THE INSTITUTIONAL WORKSHOP.

### HOSPITAL MEETINGS, &C.

### ROYAL HOSPITAL FOR DISEASES OF THE CHEST.

THE Right Honourable the LORD MAYOR (Sir John Voce Moore) presided on Wednesday, 15th inst., at the eighty-fifth annual court of Governors of the Royal Hospital for Diseases of the Chest, City Road, among those present being Sir James Whitehead, Sir Thomas de la Rue, the Hon. Lionel Ashley, and Colonel Makins.

The report and accounts were presented and adopted *nem. con.* From these it appeared that the ordinary income for the year was £8,200, and the ordinary expenditure £8,124. Legacies amounted to £545, and building improvements cost £356. The festival dinner collection realised £4,630, the largest sum ever collected for the charity on such an occasion. Donations show an increase of £3,054 on the year, but the receipts from legacies were £1,000 below the average for the past eight years. The total number of in-patients treated during 1898 was 739, as compared with 740 in 1897; the daily average of beds occupied being 57 and 58 respectively. The attendances of out-patients numbered 26,525 in 1898 as compared with 24,932 in 1897. The death of the Rev. J. H. Rose, for many years chairman of the House Committee, was referred to with deep regret, as was also the resignation for reasons of health of Miss Agnes Megginson, the matron; the place of the latter has been filled by the appointment of Miss Kate Synge.

In moving a resolution of thanks to the president (Lord Rothschild) and the other officers, the Hon. LIONEL ASHLEY referred to the fact that as regards income and expenditure they had usually to reckon upon a dead weight of some £5,000 a year, which had to be met by legacies and donations. It would be a great encouragement if they could lessen if not remove this by a large increase in their regular subscriptions.

Sir JAMES WHITEHEAD moved the re-election of the retiring members of the committee and the confirmation of the election of Sir Patteson Nickalls. He pointed out that the population of London and the suburbs was continually increasing, but that though a most laudable effort was being made by the Prince of Wales's Fund and by the Hospital Saturday and Sunday Funds to provide hospitals with the means of carrying on their work, yet the two latter at least were practically stationary. He deplored the failure of the working classes to render as much assistance as ought to be looked for from them considering how largely they benefited by the institutions. He believed that not one in twenty of them subscribed a single sixpence. He hoped for a large increase through the penny collections in workshops.

In seconding this resolution Dr. WHITE mentioned as evidence of the good work done by the council that during the year they had got a pathological laboratory, which had been badly wanted, and that the salaries of the nurses had been increased.

Sir THOMAS DE LA RUE proposed a vote of thanks to the medical officers, and said that in addition to their invaluable professional work they also devoted a great deal of time to collecting funds—Dr. White alone having raised something like £1,000 during the past ten years.

Dr. HENSLEY returned thanks for the vote. Referring to the growing feeling in favour of the treatment of tuberculosis in the purer air of the country, he said that should this be carried out there would still be plenty of work for the hospital in the treatment of other diseases of the chest.

A cordial vote of thanks to the Lord Mayor closed the proceedings.

### DENTAL HOSPITAL OF LONDON.

MR. ALDERMAN AND SHERIFF ALLISTON took the chair at the forty-first annual meeting of the Dental Hospital of London. The fund for the new building, which is now in course of erection on the site next to the present premises, and which it is hoped will be completed in about twenty months, occupied the first place in the report presented by the committee. A special appeal is made for at least £3,000 to be raised during the current year to place the building fund in a satisfactory position. £980 was transferred from the General to the Building Fund during last year. The ordinary income for the year amounted to £3,118, and the ordinary expenditure to £2,038. Added to these there were receipts £1,078, and payments £1,046 on dental appliance account. The annual subscriptions were £1,306, as against £1,226 in 1897. The committee mention that they are willing to find some other use for the portion of the site acquired for the new building unfortunately occupied by a public-house as soon as the liberality of the subscribers may put it in their power to do so. There can be no question as to the undesirability of a charity deriving income from this class of property. The Medical Committee reported that, in spite of the present limits of accommodation, there is again an increase in the annual number of cases treated and operations performed, these numbering 63,298, as against 62,512 in 1897. The report and accounts were unanimously adopted, as were also votes of thanks to the medical and other officers, and to the chairman for presiding.

### ST. MARY'S HOSPITAL, PADDINGTON.

THE forty-eighth annual meeting of St. Mary's Hospital, Paddington, was held on Friday last, 17th inst., Colonel STANLEY BIRD, chairman of the Board of Management, presiding. Among the governors present were Colonel Bannerman, Colonel Blair, Dr. Stamford Felce, Mr. Alfred Scorer, Mr. Edward Smith, and Mr. Parker Young.

From the report and accounts presented it appeared that the ordinary income was £11,913, and the ordinary expenditure £23,964. As in 1897, the hospital was indebted to legacies—which this year amounted to no less than £15,523—for tiding over what would otherwise have been a very bad year. Only £1,848 was added to the reserve fund. This result was partly due to the fact that the expenditure was very high. The causes of this, however, were largely of a temporary character, though a good many are permanent. The new out-patient department, for example, being much larger, requires a larger staff, and entails heavier expense for rates, heating, attendance, and so forth. The nursing staff also constantly tends to increase. Nearly £1,000 was spent on refurnishing the nurses' dormitories and installing a new system of gas lighting there. Endowment donations of £2,750 were received during the year, but of the 281 beds in the hospital only 16 are thus guaranteed, but it is noted with satisfaction that all but one of these have been endowed within the past ten years. A grant of £1,000 was made from the Prince of Wales's Fund, after a thorough inspection by the visitors, their report being of a complimentary character. Such defects as they noted were due solely to want of funds. From the Hospital Sunday Fund £318 less was received than in 1897; from the Saturday Fund the award was £65 higher. As foreshadowed in last year's report, a loan of £19,650 was obtained from the bankers to complete the basement of the Clarence Memorial wing as an out-patients' department. This is found to be very commodious, and to fulfil all the expectations entertained with regard to it. It being found that the interest on this loan exceeded that which the hospital was receiving on some of its investments, it was decided to sell out sufficient to repay the loan, any money hereafter raised for the extension to be devoted in the first instance to replacing the amount in the general fund of the hospital. The completion of the basement implies that the most costly part of the extension has been done. To finish the building and restore the capital account to its former position will require a sum of £70,000. It is of urgent importance that this work should be done without delay. In the last report it was announced that an inquiry officer had been appointed to investigate and report to a special committee on the pecuniary position of persons applying for free out-patient relief with a view to the prevention of abuse. During 1898, out of 12,562 cases, 1,069 were selected as calling for inquiry, with the result that 335, or less than 2⅔ per cent. of the whole, were deemed unsuitable, demonstrating that the charity has not been abused on a large scale. Every effort has been made to carry out the inquiry without hurting the feelings of the subjects of them or provoking friction. The report was adopted *nem. con.* It was mentioned that arrangements had been made for establishing centres so as to facilitate a systematic attempt to collect the funds required for the extension plans, and that already a considerable sum had been promised.

### WEST LONDON HOSPITAL.

The annual meeting of the governors of the West London Hospital was held on Wednesday, 15th inst., in the board-room of the institution, Hammersmith Road, Mr. W. Bird, the chairman of the Board of Management, presiding. The report presented stated that two of the three new wards of the additional wing in Wolverton Gardens had been opened on December 7th last by the Princess Louise. The fifty-two cots and beds of these wards were not brought into actual use until January 2nd last, although the additional nursing staff entered into residence in November. Great as was the improvement with respect to the accommodation of the nurses, they would not consider perfection had been reached until funds enabled them to carry out a further portion of the entire extension scheme. Allusion was also made to the nurses' home, for which purpose about £9,000 is required, but special stress was laid upon the fact that the entire scheme of extension would involve an expenditure of something like £54,000, in addition to the £9,000 already named. Thus, it was claimed, £63,000 must be forthcoming before the hospital could perform thoroughly and efficiently the duties which the needs of the vast district require. The pathological department had been reorganised, and by the energy of the medical staff the West London Post-Graduate College had been instituted in connection with the hospital, 130 students having been already enrolled. The West London Medico-Chirurgical Society, now numbering 550 members, still maintained its headquarters at the hospital. A scheme of distribution of the beds among the members of the medical staff having charge of in-patients had been adopted. The cases treated numbered 1,606 for the year, against 1,683 during 1897, the deaths being 138, against 158. The total cost of the in-patients was £5,680, and of the out-patients £1,241. The ordinary income was £6,199, and the ordinary expenditure £6,922, the extraordinary income being £2,948, and the extraordinary expenditure £496, the cash in hand at December 31st being £3,327.

The Chairman, in moving the adoption of the report, pointed out that they had only received £500 from the Prince of Wales's Hospital Fund, whilst their various new works left them with £9,000 owing for bricks and mortar. They felt very disappointed with the £500 from the Prince of Wales's Fund, whilst at the same time they had been asked to lay out several thousands of pounds.

Mr. A. B. Watson seconded the motion for the adoption of the report, which was carried.

The Chairman announced that the maintenance of the two new wards would cost them £3,000 a year.

### POPLAR HOSPITAL FOR ACCIDENTS.

Cheerfulness was the note both of the meeting of the Poplar Hospital and of the report and accounts presented to it. The Hon. Sydney Holland, the chairman of the General Committee, presided, and in moving the adoption of the report, said that 1898 had been an exceptionally good year for them. They had received no less than £10,000, and had spent only £9,600. That was without counting the balance of £3,500 brought forward. They did not owe a penny, and they had built a laundry at the end of their garden which would be of great advantage to them. But beyond being sound financially, they had a good record in their real work of relieving human pain and misery. They had treated 993 in-patients and 20,000 out-patients—practically 21,000 accidents had been dealt with during the year—a record of which he thought they might well be proud. And not only had there not been a single complaint, but they were constantly getting grateful letters and visits from old patients and their friends. There had been 47 fewer in-patients than in 1897, and it might be expected that the expenditure would show a corresponding decrease, but a large proportion had stayed a long time, and since such patients ate more in proportion than those only in a short time, they were more expensive to keep. The patients in the isolation block also had cost more to keep and nurse than those in the hospital. They continued to be better supported by working men than any other hospital in London in proportion to their size. Having referred to the recent explosion at Barking, from which ten men were brought into the hospital, the Chairman concluded by summarising the work of the hospital for the past seven years as follows: A new hospital has been built at a cost of £40,000. The work of the hospital has doubled. The treatment of the patients has been improved. The staff have greater privileges. The cost of maintenance has been decreased for the work done. An addition has been made to the reserve fund of £8,000, and the hospital is absolutely free from debt. The report having been adopted, the committee was re-elected, with the addition of Mr. Zincman, and votes of thanks brought the proceedings to a close.

### THE BRITISH LYING-IN HOSPITAL, ENDELL STREET.

Many improvements are apparent in this old and useful hospital. Much in progress still remains to be finished, so only what has been already done can be mentioned. The number of beds has been raised from 16 to 20 by putting one extra bed—that is five instead of four—in each ward. No. 220, Endell Street, formerly sub-let, has been incorporated with the hospital. By this means an additional staircase is available for paying patients and the nurses, whilst the matron has a pleasant sitting-room. A lift has been put in at a cost of £50, and a new kitchen has been constructed in a disused room in the basement. Everywhere there are evidences of cleaning and repairs. One ward is just quit of house decorators. The walls are painted two shades of a cheerful green, whilst new beds, coverlets, blinds, linoleum (not yet laid), a cupboard in which there is a shelf for each patient's use, give an excellent idea of the spick and span appearance the hospital will present when the improvements are completed. A new ward table is also promised.

The report is satisfactory in all but two items. The one that the number of subscribers and amount of subscriptions have slightly decreased during last year; and the other that the subscribers, especially the lady subscribers, do not take the personal interest in the hospital that would, in the expressed opinion of the chairman, be so advantageous to the institution. For the benefit of those who would like to know, it may be here mentioned that the matron, Miss Cook, is always at home on Wednesday afternoons (as well as at other times), and ready to show the hospital to any of the contributors.

The Chairman (Mr. C. E. Farmer) at the annual meeting held at the hospital on the 21st inst. drew special attention to the accounts. The annual subscriptions amounted to £312 (of which sum £25 was received from contributions in arrears), against £295 for 1897. The Hospital Sunday Fund gave £18 (less than in 1897), the Hospital Saturday Fund £40 (also less than 1897); whilst the Prince of Wales's Hospital Fund doubled its grant. The Nursing Institution has increased greatly. The nurses' fees are £221, against £38 in the previous year; the midwifery pupils' fees £57, against £44, whilst the cost of maintenance per head, including nurses, domestics, and patients, has been reduced to an average of 11½d. per day. The ordinary expenditure was twopence short of £1,830; the extraordinary £924 11s. 8d.; leaving a deficit in the year's account of £505. The number of in-patients reached 269, an increase of 82 on the previous year, whilst the number of out-patients were 411, an increase of 21.

The changes in the officers are as follows: Mr. A. H. P. Strickland has been elected treasurer in the place of the late Mr. Charles Hoare; Lord Kinnaird has been elected vice-president in the stead of the late Marquis of Exeter, K.G.; Sir William MacCormac, Bart., F.R.C.S., has accepted the post of consulting surgeon, held by the late Sir Spencer Wells; and Mr. H. Fitzherbert Wright has been elected to the Board of Management.

### METROPOLITAN HOSPITAL, KINGSLAND ROAD.

The sixty-third annual meeting of the Metropolitan Hospital, Kingsland Road, was held on Monday last, 20th inst., Mr. Charles J. Thomas, the chairman of the Committee of Management, presiding.

The report presented showed that the total receipts for 1898 amounted to £12,605, an increase of £4,460 on the previous year. Of this £5,117 was legacies. The ordinary expenditure was £9,243, as against £9,490 in 1897. The extraordinary expenditure was £837 as compared with £145—accounted for by the fact that the greater part of the hospital was re-painted during the year. The debt to tradesmen has been reduced from £7,176 to £4,941. The number of beds available in 1898 was 72 for six months and 96 for six months, the increase being in consequence of 24 beds having been placed at the disposal of the Shoreditch Guardians, thus increasing the daily average number of beds occupied to 78 as compared with 70 in 1897. The average stay of each in-patient was 29½ days as against 24½, those admitted from the Shoreditch Infirmary staying longer than others; 962 in-patients were treated during the year, and 30,017 out-patients—the attendances of the latter numbered 99,331—6,162 visits were made at the homes of members of the Provident Department by the medical officers, and 26,982 attendances were made at the hospital, the membership of the department numbering 4,424 at the end of the year. A letter of thanks was received from the Town Clerk of Maidstone for the use of the Convalescent Home during the typhoid epidemic by the Maidstone authorities. Miss Mabel Cave having resigned her post as matron to take up a similar position at the Westminster Hospital, Miss J. C. Bennet was appointed in her place. The committee report that the nursing hours compare favourably with those in force at other hospitals. The roadway in front of the hospital will shortly be paved with wood—a concession from the Local Board and the Tramways Company for which the committee are very thankful. Mr. H. L. Webster Lawson, L.C.C., has promised to preside at the festival dinner on Monday, 24th April, at the Whitehall Rooms.

In moving the adoption of the report, the Chairman expressed the gratification that all must feel that their income had made such a happy bound from £8,000 to well over £12,000, but they must recollect that since the increase was chiefly from legacies it meant that a certain number of well-wishers would help them no more, and, therefore, do their utmost to get new friends and subscribers. He mentioned that when the Prince of Wales's Fund sent them £500 as a result of the visit of two members of that body, Mr. Sandeman, one of the visitors, sent also a cheque for £50 on his own private account. He also announced that arrangements were being made for the formation of a ladies' association in connection with the hospital. The inaugural meeting was to take place in May, and great things were hoped from the new departure.

Mr. Ernest Flower, M.P., seconded the resolution, which was carried, and the meeting separated after passing votes of thanks to the staff and to the chairman for presiding.

